# Reparo aberto de aneurisma de aorta toracoabdominal: atualização da abordagem multimodal

**DOI:** 10.1590/1677-5449.071317

**Published:** 2017

**Authors:** Roberto Chiesa, Germano Melissano, Enrico Rinaldi

**Affiliations:** 1 San Raffaele Scientific Institute, Vita-Salute University, Faculdade de Medicina, Cirurgia Vascular, Milão, Itália.

A cirurgia convencional de aneurismas de aorta toracoabdominal (AATA) evoluiu de maneira significativa ao longo das últimas décadas graças a refinamentos técnicos, especialmente com relação à proteção visceral. Entretanto, apesar das estratégias adjuntivas, as taxas de morbidade e mortalidade ainda não são negligenciáveis.

Com o intuito de planejar a melhor modalidade terapêutica para cada paciente, imagens precisas devem ser obtidas e processadas. O método de imagem de escolha para efetuar esse planejamento é a angiotomografia computadorizada *multislice*. Além de definir os diâmetros e a extensão do aneurisma, a análise dos vasos envolvidos inclui características importantes como calcificação e presença de trombo, possíveis variações anatômicas e patência dos vasos. No momento, o diâmetro aórtico é o melhor preditor do risco de rotura^1^, e os pacientes portadores de AATA devem ser considerados para cirurgia eletiva se o diâmetro aórtico exceder 6,0 cm, ou com diâmetros menores no caso de pacientes com dissecção crônica ou doenças do tecido conjuntivo.

Com o envelhecimento da população, observa-se um aumento do número de comorbidades nos pacientes portadores de AATA. Uma adequada avaliação pré-operatória das funções cardíaca, pulmonar e renal e o conhecimento minucioso da anatomia vascular cerebral e da medula espinhal são úteis durante a avaliação do risco operatório e para o planejamento da melhor estratégia cirúrgica.

O ecocardiograma transtorácico pré-operatório é um satisfatório método de rastreamento não invasivo que avalia tanto a função valvular quanto a biventricular. A angiotomografia de artérias coronárias emergiu como um método não invasivo capaz de avaliar a doença arterial coronariana, que deve ser realizado de rotina em pacientes portadores de AATA assintomático. Em caso de doença arterial coronariana importante, o cateterismo cardíaco deve ser realizado, e as lesões significativas devem ser tratadas com angioplastia transluminal percutânea e colocação de stent anteriormente ao reparo cirúrgico do AATA. Deve-se evitar, quando possível, a inserção de stents farmacológicos que podem exigir dupla antiagregação plaquetária por período prolongado. A revascularização do miocárdio limita-se a pacientes selecionados, portadores de lesões coronarianas severas de alto risco e inadequados para angioplastia transluminal percutânea.

A função renal constitui um preditor estabelecido da evolução pós-operatória. Com base na avaliação da taxa de filtração glomerular estimada, a doença renal crônica demonstrou ser um importante preditor de morte tanto após a correção convencional quanto após a correção endovascular dos aneurismas torácicos, até mesmo em pacientes sem evidências clínicas de doença renal pré-operatória^2^.

A avaliação da função pulmonar com gasometria arterial e espirometria deve ser realizada em todos os pacientes que serão submetidos a correção convencional do AATA. A tomografia cerebral e a avaliação neuropsicológica são realizadas eletivamente. Anomalias anatômicas ou doenças cerebrais que possam contraindicar a drenagem liquórica devem ser identificadas. A estratégia cirúrgica deve ser explicada no pré-operatório para todos os pacientes eletivos, e o consentimento informado deve ser obtido.

No tocante à estratégia cirúrgica, o paciente é posicionado em decúbito lateral direito sob um colchão de areia com os ombros a 60 graus e o quadril flexionado posteriormente a 30 graus para permitir acesso total ao tórax esquerdo, ao abdome e à região inguinal esquerda. Um colchão térmico de água circulante com permutador de calor é colocado entre o colchão de areia e o paciente, com o intuito de auxiliar na manutenção da temperatura corporal.

As incisões para toracotomia variam em comprimento e nível, de acordo com a extensão do aneurisma. A prática padrão é a secção deliberada da costela do espaço intercostal incisado posteriormente, para evitar fraturas espontâneas com margens agudas irregulares. A paralisia do hemidiafragma esquerdo causada por sua divisão radial no hiato aórtico pode contribuir significantemente para insuficiência respiratória pós-operatória; portanto, a secção circunferencial limitada do diafragma é rotineiramente realizada, com preservação do centro frênico. Sob condições anatômicas favoráveis, a secção circunferencial do diafragma demonstrou reduzir o tempo de desmame respiratório^3^.

O clampeamento da aorta torácica descendente promove relevantes alterações hemodinâmicas, incluindo importante aumento da pós-carga e da isquemia visceral. A técnica para perfusão aórtica distal com circulação extracorpórea, denominada *left heart bypass* (LHB), provou ser extremamente útil durante o reparo aórtico. O fundamento do LHB consiste em fornecer fluxo sanguíneo para medula espinhal, vísceras e rins durante o período de clampeamento aórtico, associado à redução na hipertensão proximal e na pós-carga cardíaca. A veia pulmonar esquerda é comumente canulada para drenagem do sangue arterial, que será reinfundido através de bomba centrífuga (BioPump® - Medtronic Biomedicus, Inc., Minneapolis, MN, USA) na aorta subdiafragmática ou na artéria femoral comum esquerda^4^.

Após o início do LHB, a aorta é cuidadosamente clampeada em seu segmento mais apropriado, imediatamente após a artéria subclávia esquerda (ASE) ou entre a artéria carótida comum esquerda e a ASE quando o aneurisma envolver a aorta descendente proximal. Uma vez que a porção proximal da aorta estiver isolada entre os clampes, a aorta torácica descendente é seccionada e isolada do esôfago. O sangramento proveniente das artérias intercostais proximais é controlado com suturas reforçadas com *pledgets*. A porção proximal da prótese é anastomosada na aorta torácica descendente através de sutura contínua, com uso de fio monofilamentar de polipropileno 2/0 ou 3/0. A anastomose é reforçada com faixas de teflon ou *pledgets*.

Exceto em pacientes portadores de *shaggy aorta* (aorta com lesões ulcerativas e trombos), em que a aorta é clampeada logo acima e abaixo do aneurisma a fim de reduzir o risco de embolia, rotineiramente o clampe aórtico distal é removido e reposicionado na aorta torácica distal acima do tronco celíaco (clampeamento sequencial), e o aneurisma é aberto.

As artérias segmentares principais e patentes de T7 a L2 são identificadas e temporariamente ocluídas com cateteres de Pruitt para evitar o fenômeno de roubo de fluxo. Com base na monitorização neurofisiológica, as artérias intercostais devem ser reimplantadas a partir de incisão protética lateral e confecção de *patch* através da *island technique*. As artérias intercostais principais também podem ser reimplantadas através da interposição de enxerto protético.

O clampe distal é então posicionado abaixo das artérias renais, e o aneurisma é aberto. A perfusão hemática visceral é mantida pela bomba com auxílio de cateteres de oclusão-irrigação introduzidos seletivamente no tronco celíaco e na artéria mesentérica superior. A perfusão renal fria e seletiva é realizada: inicialmente, uma solução cristaloide com manitol e esteroides a 4 °C era utilizada; entretanto, seguindo as evidências de melhor preservação da função renal com a perfusão com custodiol (histidina-triptofano-quetoglutarato)^5^, recentemente introduzimos o uso rotineiro dessa solução em nossos pacientes.

Se uma estenose serrada for observada antes da colocação do cateter de oclusão-irrigação, a colocação de um stent ostial pode ser efetuada pela inserção de um stent expansível por balão de tamanho apropriado dentro da artéria. Uma incisão lateral é confeccionada no enxerto protético, e o tronco celíaco, e a artéria mesentérica superior e as artérias renais são reimplantadas através de *patch* de Carrel. A fim de reduzir a quantidade de aorta nativa, a artéria renal esquerda é habitualmente reimplantada separadamente através da interposição de um enxerto de dácron.

Por fim, é realizada a anastomose terminoterminal com a porção aórtica distal, e o último clampe é removido. Em alguns casos (AATA de tipo I), as artérias viscerais podem ser incorporadas em uma anastomose distal biselada.

No caso de reimplante seletivo dos vasos viscerais ou renais, a prótese vascular Gore Hybrid® (Gore Hybrid Vascular Graft – GHVG, Flagstaff, Arizona, EUA) pode ser utilizada. O segmento de stent constrito do GHVG é minuciosamente inserido em 2-3 cm dentro da luz arterial, através do fio-guia, em relação aos ramos colaterais, e então liberado.

A pós-dilatação do *stent* é realizada em todos os casos após a liberação do segmento distal do GHVG com auxílio de balão não complacente de 5-6 mm. O *stent* é então mantido em posição com pontos simples de polipropileno monofilamentar, confeccionados circunferencialmente ao vaso. Por fim, a anastomose proximal do GHVG no enxerto aórtico principal é realizada de maneira usual após a secção da porção proximal da prótese, sem stent, com comprimento apropriado^6^.

A lesão da medula espinhal durante os procedimentos toracoabdominais ocorre principalmente devido a um insulto isquêmico. Entretanto, sua fisiopatologia é extremamente complexa e ainda muito pouco compreendida. Como recentemente publicado, a incidência de isquemia medular após a cirurgia convencional de AATA, em particular os aneurismas extensos do tipo II, ainda constitui um problema, com uma taxa de apresentação de 9,6%^7^. A isquemia medular pode resultar em deficiência física devastante e em sobrevida muito reduzida no seguimento pós-operatório^8^.

A proteção da medula espinhal representa uma etapa fundamental da estratégia pré-operatória e intraoperatória do tratamento do AATA, e uma abordagem multidisciplinar é necessária. O tempo de clampeamento aórtico constitui o preditor pós-operatório mais importante de paraplegia na cirurgia convencional. Na tentativa de reduzir o tempo de isquemia, diversas técnicas de perfusão aórtica distal associadas ao clampeamento sequencial foram introduzidas para permitir a perfusão dos vasos nutridores da medula espinhal durante o período de clampeamento aórtico. O efeito protetor do LHB associado ao clampeamento aórtico sequencial, em comparação à realização da técnica *clamp and sew*, já foi publicado^9^.

A importância do reimplante das artérias intercostais principais na redução do risco de paraparesia/paraplegia pós-operatória é bem demonstrada^10^. Esse procedimento, entretanto, é demorado, e um segmento extenso de aorta reimplantado pode tornar-se propenso a dilatação futura; portanto, recomendamos evitar reimplantes desnecessários, especialmente em pacientes portadores de doenças do tecido conjuntivo. Avanços recentes nos métodos de imagem podem desempenhar um papel relevante no planejamento do reimplante seletivo das artérias intercostais principais^11^.

A otimização da perfusão medular, que eleva a pressão arterial sistêmica e reduz a pressão do líquido cefalorraquidiano, é também utilizada para a prevenção e o tratamento da isquemia medular. A estabilidade hemodinâmica é muito importante, e a pressão arterial média deve ser mantida, em geral, acima de 70 mmHg.

A pressão do líquido cefalorraquidiano eleva-se imediatamente após o clampeamento aórtico e após a isquemia medular. Juntamente à diminuição da pressão de perfusão medular, esse mecanismo pode ser uma das principais causas de isquemia medular. A pressão do líquido cefalorraquidiano pode ser facilmente monitorizada, e a drenagem do liquor com o intuito de reduzir sua pressão a níveis abaixo de 10 cmH_2_O é amplamente praticada. Uma revisão da literatura confirmou a eficácia da drenagem do líquido cefalorraquidiano na prevenção e no tratamento da isquemia medular após o tratamento do aneurisma torácico e do AATA^12^. A drenagem do líquido cefalorraquidiano pode ser realizada com segurança com o uso do LiquoGuard® (Möller Medical GmbH, Fulda, Alemanha), um novo dispositivo para drenagem liquórica controlada e contínua, projetado para manter a pressão do líquido cefalorraquidiano próxima aos valores desejados. Isso evita a drenagem desnecessária e permite a monitorização simultânea da pressão liquórica e sua drenagem ativa^13^.

A detecção precoce da isquemia medular é essencial para permitir uma intervenção imediata antes que a isquemia evolua para infarto e paraplegia permanente. A monitorização neurológica da função medular pode ser realizada em pacientes anestesiados com a análise dos potenciais evocados somatossensoriais e dos potenciais evocados motores ou de ambos^14^. Os registros da linha de base são obtidos após a indução anestésica e intubação, a fim de garantir as medições quando o estado anestésico de estabilidade for atingido e o efeito da dose inicial do relaxante muscular desaparecer. Os potenciais evocados motores são verificados intermitentemente até que a aorta seja clampeada e a cada 5 minutos durante e após o clampeamento aórtico. A perda persistente dos potenciais evocados motores em três estimulações consecutivas é considerada significativa e utilizada como um “sinal de alarme intraoperatório” para manobras perioperatórias adicionais.

Na última década, houve uma melhora significativa na correção convencional do AATA, o que levou a uma redução na mortalidade precoce e nas complicações neurológicas, com uma mortalidade média em 30 dias de 7% e uma taxa de isquemia medular média de 7,5%^15^. Além disso, nos últimos anos, em centros de excelência e alto volume, métodos auxiliares para proteção renal reduziram a incidência de insuficiência renal após o reparo convencional do AATA^5^.

A cirurgia convencional do AATA, graças às melhorias técnicas, é atualmente o padrão-ouro em pacientes adequados. A seleção dos pacientes deve ser baseada em uma cuidadosa avaliação pré-operatória e de risco cirúrgico. A correção cirúrgica do AATA é melhor realizada em centros de alto volume por cirurgiões experientes e equipes multidisciplinares qualificadas.
