# Supplementary material for: Reparo aberto de aneurisma de aorta toracoabdominal: atualização da abordagem multimodal
Source: J Vasc Bras. 2017 Jul-Sep;16(3):183–6. [Article in Portuguese] doi: 10.1590/1677-5449.071317 (PMC5868932; doi:10.1590/1677-5449.071317)
Supplement: Translation

# Translation: Open TAAA repair: updates on multimodal approach

**Authors:** Roberto Chiesa, Germano Melissano, Enrico Rinaldi

**Affiliations:** 1 San Raffaele Scientific Institute, Vita-Salute University, School of Medicine, Vascular Surgery, Milano, Italy.

Open surgical repair of thoracoabdominal aortic aneurysms (TAAA) has evolved significantly over the last decades thanks to technical improvements, especially in the area of organ protection. However, despite adjunctive strategies, morbidity and mortality rates are still not negligible.

In order to plan the best possible treatment modality for every patient, accurate imaging must be obtained and processed. The preferred imaging method is multidetector computed tomography (CT) scan. As well as defining diameters and extension of the pathology, analysis of vessels includes characteristics of calcification and thrombus, possible anatomic variations, and patency of vessels. At present, aortic diameter is the best criterion for predicting the risk of aortic rupture^1^ and patients with TAAA can be considered for elective surgery if the aortic diameter exceeds 6.0 cm, or less in case of patients with chronic dissection or a connective tissue disorder.

As the number of elderly people in the population has increased, so has the number of patients with TAAA associated with comorbidities. An adequate pre-operative assessment of cardiac, pulmonary, and renal function and an accurate knowledge of cerebral and spinal cord vascular anatomy are useful when evaluating operative risk and planning the best operative strategy.

Pre-operative trans-thoracic echocardiography is a satisfactory noninvasive screening method that evaluates both valvular and biventricular function. Computed tomographic coronary angiography has emerged as a less-invasive method for assessing coronary artery disease and is routinely performed in patients with asymptomatic TAAA. In case of severe coronary artery disease, a coronary angiography is then performed and significant lesions are treated with percutaneous transluminal angioplasty prior to aneurysm repair, possibly avoiding the use of drug-eluting stents that would require prolonged double antiplatelet therapy. Surgical myocardial revascularization is limited to selected patients with severe high-risk coronary lesions that are not suitable for percutaneous transluminal angioplasty.

Renal function is an established predictor of postoperative outcome. Based on eGFR assessment, chronic kidney disease has been shown to be a strong predictor of death after thoracic aneurysm repair for both open and endovascular procedures, even in patients without clinical evidence of preoperative renal disease.^2^

Evaluation of pulmonary function with arterial blood gases and spirometry is performed for all patients undergoing open TAAA repair.

A CT brain scan and neuropsychological evaluation are performed in the elective setting. Anatomical anomalies or cerebral diseases that may contraindicate the use of Cerebrospinal fluid (CSF) drainage should be identified.

Surgical strategy is preoperatively explained to any elective patient and informed consent is obtained.

With regard to surgical strategy, the patient is positioned in a right lateral decubitus over a beanbag with the shoulders at 60° and the hips flexed back to 30° to access the entire left thorax, the abdomen, and the left groin. A circulating water mattress with heat exchanger is placed between the beanbag and the patient, in order to facilitate body temperature management.

The thoracotomy incision varies in length and level, depending on the extent of the aneurysm. Our standard practice is to deliberately cut the rib of the incised intercostal space posteriorly, in order to avoid spontaneous fractures with irregular acute margins. Since paralysis of the left hemidiaphragm produced by its radial division to the aortic hiatus may contribute significantly to postoperative respiratory failure, a limited circumferential section of the diaphragm is routinely carried out, sparing the phrenic center. Under favorable anatomic conditions, this has been shown to reduce respiratory weaning time.^3^

Cross-clamping of the descending thoracic aorta leads to several hemodynamic disturbances, including severe afterload increase and organ ischemia. The technique for distal aortic perfusion with a left heart bypass (LHB) has proved to be extremely useful during aortic repair. The rationale of LHB is providing flow to the spinal cord, viscera, and kidneys during the aortic cross-clamp period, together with reduction of proximal hypertension and afterload to the heart. The left pulmonary vein is usually cannulated to drain arterial blood, which is reinfused through a centrifugal pump (BioPump® - Medtronic Biomedicus, Inc., Minneapolis, MN, USA) into the subdiaphragmatic aorta or the common left femoral artery.^4^

After the LHB is started, the aorta is gently cross-clamped at the most appropriate site, either immediately after the left subclavian artery (LSA) or between the left common carotid artery and LSA when the aneurysm involves the proximal descending aorta. Once the proximal aspect of the aorta is isolated between clamps, the descending thoracic aorta is transected and separated from the esophagus. Bleeding proximal intercostal arteries are oversewn with pledgeted sutures. The proximal end of the graft is sutured to the descending thoracic aorta using 2/0 or 3/0 monofilament polypropylene in a running fashion and the anastomosis is reinforced with Teflon felt or pledgets.

Except for patients with “shaggy aorta” in which the aorta is clamped just above and below the aneurysm in order to reduce the risk of embolism, routinely, the distal aortic clamp is removed and reapplied onto the distal thoracic aorta above the celiac axis (sequential cross-clamping) and the aneurysm is opened.

Critical patent segmental arteries from T7 to L2 are identified and temporarily occluded with Pruitt catheters to avoid the blood-steal phenomenon. Depending on neurophysiologic monitoring, the intercostal arteries may be reattached to a tailored side-cut in the graft by means of an island technique. Critical intercostal arteries may also be selectively reattached with a tube graft interposition.

The distal clamp is then moved below the renal arteries and the aneurysm is opened. Visceral hematic perfusion is maintained by the pump with irrigation-occlusion catheters introduced selectively into the celiac trunk and the superior mesenteric artery. Selective cold perfusion of renal arteries is performed: initially a crystalloid solution with mannitol and steroids at 4 °C was used; however, following evidence of improved kidney preservation by perfusion with Custodiol (histidine-tryptophane-ketoglutarate),^5^ we recently introduced routine use of this solution in our patients.

If a tight stenosis is encountered, before placing the irrigation occlusion catheter, orificial stenting may be accomplished by direct placement of an appropriate-sized balloon expandable stent within the artery. A side cut is tailored in the graft and the celiac trunk, superior mesenteric artery, and renal arteries are reattached by means of a Carrel patch. To reduce the amount of native aorta, the left renal artery is usually reattached separately via a Dacron graft interposition.

Finally, an end-to-end anastomosis with the distal aorta is performed and the last clamp removed. In some cases (Extent I) the visceral arteries can be incorporated in a beveled distal anastomosis.

In case of selective reattachment of visceral or renal vessels, a gore hybrid vascular graft (GHVG - Hybrid Gore, Flagstaff, Arizona USA) can be used. The constrained stented segment of the GHVG is gently placed over-the-wire into the artery for 2 to 3 cm, with respect of collateral branches, and then deployed. Stent post-dilation is performed in all cases after GHVG distal segment deployment with a 5- to 6-mm noncompliant balloon. The stent is then sewn in place with single circumferential monofilament polypropylene stitches. Finally, the proximal anastomosis to the main aortic graft is completed in the usual fashion after the proximal unstented section of the graft is cut to the proper length.^6^

Spinal cord (SC) damage during thoracoabdominal procedures is mainly due to ischemic insult. However, its pathophysiology is much more complex and still poorly understood. As recently reported, the incidence of SC ischemia following open TAAA repair, particularly in extensive type II aneurysms, still remains a problem with a rate of presentation of 9.6%.^7^ Spinal cord ischemia may result in devastating physical disability and a much reduced survival at follow-up.^8^

Spinal cord protection represent a fundamental part of the pre-operative and intra-operative TAAA management strategy, and a multimodal approach is advocated. Aortic cross-clamp time is the most significant predictor of postoperative paraplegia in open surgery. In an attempt to reduce ischemia time, several techniques for distal aortic perfusion associated with sequential clamping were introduced to allow perfusion of vessels feeding the SC while the aorta is clamped. It has been reported that left heart bypass combined with sequential clamping has a protective effect, compared with the simple “clamp and sew” technique.^9^

The importance of reattachment of critical intercostal arteries to reduce the risk of postoperative paraparesis/paraplegia is well demonstrated.^10^ This procedure, however, is time-consuming and a large aortic reattached island may be prone to further dilatation and so it would be probably be better to avoid unnecessary reimplantations, especially in patients with connective tissue disorders. Recent advances in imaging may play a role in planning selective reimplantation of important SC arteries.^11^

Optimizing SC perfusion by raising arterial systemic blood pressure and reducing CSF pressure is also used for prevention and treatment of SC ischemia. Hemodynamic stability is very important and in general mean arterial pressure should be maintained over 70 mmHg.

Cerebrospinal fluid pressure rises immediately after cross-clamping and after SC ischemia. Coupled with decreased spinal perfusion pressure, this mechanism may be one of the major causes of SC ischemia. The CSF pressure can be easily monitored, and drainage of CSF to reduce the pressure to below 10 cmH_2_O is widely practiced. A review of the literature has confirmed the effectiveness of CSF drainage to prevent and treat SC ischemia after treatment for thoracic aneurysms and TAAA.^12^ Cerebrospinal fluid drainage could be safely performed with LiquoGuard (Möller Medical GmbH, Fulda, Germany) a new device for controlled and continuous CSF drainage designed to maintain CSF pressure around the desired set values, avoiding unnecessary drainage and allowing simultaneous monitoring of CSF pressure and active drainage.^13^

Early detection of SC ischemia is critical to allow prompt intervention before ischemia evolves to infarction and permanent paraplegia. Neurologic monitoring of SC function can be performed in anesthetized patients with somatosensory evoked potentials (SSEP), motor evoked potentials (MEP), or both.^14^ Baseline recordings are obtained after induction of anesthesia and intubation in order to guarantee the measurements when a steady anesthetic state has been reached and the effect of the initial dose of muscle relaxant has disappeared. Motor evoked potentials are checked intermittently until the aorta is cross-clamped and every 5 minutes during and after cross-clamping. Persistent MEP loss in three consecutive stimulations is considered significant and used as an “intraoperative alarm sign” for additional intraoperative maneuvers.

In the past decade, there has been a dramatic improvement in open TAAA repair, leading to reductions in early mortality and neurologic complications, with a current mean 30-day mortality of 7% and an overall mean spinal cord ischemia of 7.5%.^15^ Moreover, in recent years in high volume centers, technical adjuncts for renal protection have reduced the incidence of renal failure following open TAAA repair.^5^

Thanks to technical improvements, open surgical repair of TAAA, is currently the gold standard in fit patients. Patient selection must be based on careful preoperative assessment and risk evaluation. Surgical TAAA repair is best performed in high-volume centers by experienced surgeons and skilled multidisciplinary teams.
